# Bone Regeneration Using PEVAV/β-Tricalcium Phosphate Composite Scaffolds in Standardized Calvarial Defects: Micro-Computed Tomographic Experiment in Rats

**DOI:** 10.3390/ma14092384

**Published:** 2021-05-03

**Authors:** Mohammed Badwelan, Mohammed Alkindi, Osama Alghamdi, Abeer Ahmed, Sundar Ramalingam, Ali Alrahlah

**Affiliations:** 1 Department of Oral and Maxillofacial Surgery, College of Dentistry, King Saud University, Riyadh 11545, Saudi Arabia; malkindi@ksu.edu.sa (M.A.); oghamdi@ksu.edu.sa (O.A.); smunusamy@ksu.edu.sa (S.R.); 2 Department of Periodontics and Community Dentistry, College of Dentistry, King Saud University, Riyadh 11545, Saudi Arabia; AliAbeer@ksu.edu.sa; 3 Restorative Dental Sciences Department, College of Dentistry, King Saud University, Riyadh 11545, Saudi Arabia; aalrahlah@ksu.edu.sa; 4 Engineer Abdullah Bugshan Research Chair for Dental and Oral Rehabilitation, College of Dentistry, King Saud University, Riyadh 11545, Saudi Arabia

**Keywords:** bone regeneration, critical-size defect, beta-tricalcium phosphate, micro-computed tomography, composite scaffold

## Abstract

Bone regeneration using beta-tricalcium phosphate (β-TCP) can be practiced using a biocomposite scaffold. Poly(ethylene-co-vinylalcohol)/poly(δ-valerolactone)/β-tricalcium phosphate (PEVAV/β-TCP) composite scaffolds showed promising in vitro results. This study evaluated the bone regenerative potential of PEVAV/β-TCP biocomposite scaffolds in standardized calvarial defects in a rat model over 4 and 10 weeks. Bilateral calvarial defects (5 mm in diameter and about 1.5 mm thick, equivalent to the thickness of the calvaria) were created in 40 male Wistar albino rats. The defects were grafted with either commercially available β-TCP (positive control), PEVAV/β-TCP 70, or PEVAV/β-TCP 50, or left empty (negative control), depending on the group to which the animal was randomly assigned, to be covered before flap closure with resorbable collagen membrane (RCM). At 4 and 10 weeks post-surgery, the collected rat calvaria were evaluated using micro computed tomography (micro-CT) analysis, to assess the newly formed bone volume (NFBV), newly formed bone mineral density (NFBMD), and remaining graft volume (RGV). The results showed that calvarial defects grafted with the PEVAV/β-TCP biocomposite exhibited higher NFBV than did control defects, both at 4 and 10 weeks post-surgery. Furthermore, calvarial defects grafted with PEVAV/β-TCP 70 showed the highest NFBV among all grafting conditions, with a statistically significant difference recorded at 10 weeks post-surgery. The PEVAV/β-TCP composite scaffold showed potentiality for the regeneration of critical-sized calvarial bone defects in a rat model.

## 1. Introduction

Bone is a critical organ because of its vital role in body physiology, such as blood production, mineral storage and hemostasis, skeleton balance, and organ protection. The significance of bone becomes more evident in conditions like osteomyelitis and osteoporosis. Moreover, bone defects resulting from traumatic injuries, tumor resection, or alveolar bone resorption following tooth loss, and the resulting defects with variable width and height, represent a challenging clinical situation [1]. Despite being the gold-standard bone grafting material, autologous bone grafting is associated with increased morbidity because it requires creating a second donor site. Conversely, grafting materials, such as allografts and xenografts, imply the possibility of immune rejection and disease transmission. Hence, several synthetic bone-grafting materials have been developed and applied for osseous defect regeneration in the field of clinical dentistry and orthopedic surgery that showed significant success without the risk of disease transmission [2]. Among them, beta-tricalcium phosphate (βTCP) has generated a great interest in bone regeneration both in animals [3,4] and humans [5,6] because of its biocompatibility, similarities with natural bone minerals, and resorptive characteristics. However, commercially available βTCP scaffolds are stiff granules, offering shape differences compared with the defect and inadequate structural support and restricting the choices of clinicians regarding the customization of the scaffold consequently increase the need for resorbable collagen membranes (RCM) to ensure the stability of a particulate graft material at non-self-confined defects. A further characteristic of RCM, such as fostering hemostasis by inducing platelet attachment, emphasized its function in bone regeneration [3].

Composite scaffolds have been developed to reduce the stiffness of calcium phosphate in general, and β-TCP in particular, and offer an option to surgeons in the design of the scaffold based on the defect; this is accomplished by bonding β-TCP particles to a biodegradable polymer, to yield a structure that simulates the bone architecture. Despite the expanding investigation in the field of tissue engineering using available natural polymers (including, but not limited to, alginate, silk, collagen, and gelatin), a limited number of these polymers have been applied in scaffold synthesis, because of their low compressive strength and elastic modulus, the possibility of triggering an allergic reaction and transmit diseases, rapid resorption rate with an inability to maintain scaffold structural integrity (which does not meet the time needed to regenerate bone); consequently, scaffold structural integrity could not be maintained using these materials. In addition, their limited availability dramatically increases their price. Hence, to develop a scaffold that exhibits the essential properties and acts as a bone scaffold, in which single polymer does not inherit exclusively, combining more than one polymer (polymer blend) with the β-TCP has been the aim of many researchers toward the design of a hybrid material for application in the biomedical field, specifically in bone regeneration.

A polymer blend is a mixture of more than one polymer; this was introduced to generate new materials with optimized properties capable of overcoming the disadvantages of individual polymers. For instance, Sadiasa et al. evaluated a porous three-dimensional (3D) scaffold composed of various ratios of polycaprolactone (PCL) blended with poly l-lactic acid (PLLA) for bone tissue regeneration. The technique used to synthesize the polymer blend enables the authors to compare different polymer ratios and porosity percentages [7]. The integration of other molecules (e.g., antibiotics) is an additional advantage of developing a composite scaffold, Miyai et al. reported in 2007 that poly-e-caprolactone (PCL) and polyethylene glycol were blended with gatifloxacin and porous β-TCP [8]. Another study by Shuai et al. showed promising in vitro results, in which varying amounts of polyglycolic acid (PGA) polymer and poly-l-lactic acid (PLLA) were blended. Concomitantly, the hydroxyapatite (HAP) weight percent (wt%) remained constant. The micro-CT has additionally confirmed that after the in vivo experiment [9].

Poly(ethylene-co-vinyl alcohol) (PEVAL) is one of the most popular and flexible materials. It is a semi-crystalline random used in many medical applications because of its high gas barrier potential, biocompatibility, and non-toxicity [10]. A copolymer with safe ethylene units is liable to degradation and can undergo complete biodegradation, notably in the presence of enzymes [11]. Poly(δ-valerolactone) (PDVL) is a semi-crystalline aliphatic structure with overall similar chemical properties to those of PCL, with the exception of a less elastomeric behavior. It has good biodegradability, biocompatibility, and permeability characteristics, which render it a necessary and vital aliphatic polyester [12]. Although the scaffold composed of a of PEVAL/PDVL blend (PEVAV) reported previously by Saeed et al. [13] and showed excellent properties, the blend containing equal amounts of the two polymers exhibited the best performance, especially for cellular viability and adhesion. However, it had never been assessed as a bone scaffold until ceramic-based bone graft (β-TCP) particles were bound to the polymer blend, which improved the scaffold properties as a bone scaffold in terms of physical, mechanical, and biological characteristics, and provided an option to increase the operational freedom of surgeons and the resilience of ceramic-based bone graft substitute materials in a 3D polymer scaffold structure that mimics bone and can be shaped depending on the required application [14,15]. Nevertheless, the application of this biocomposite scaffold to an animal model is required to investigate its regenerative potential.

This study used micro-CT to prove the hypothesis that new bone formation can occur in standardized calvarial defects in a rat model over 4 and 10 weeks when grafted with PEVAV/β-TCP biocomposite scaffold compared with the untreated (empty) control. In addition, it was designed to compare the PEVAV/β-TCP 50 and 70 wt%, biocomposite scaffold to a commercially available (positive control) porous β-TCP with comparable physical properties (60% total porosity and pore size ranging from 100 to 500 µm), which is already used clinically as a bone substitute (ChronOS™).

## 2. Materials and Methods

### 2.1. Scaffold

The new PEVAV/β-TCP biocomposite scaffold was obtained using the solvent casting/particulate leaching technique. Briefly, 50 and 70 wt% of β-TCP particles were added to a predetermined amount of PDVAL/PEVAL (1:1 wt% ratio), which were all dissolved in a minimum of DMF at 80 °C with continuous stirring, until complete dissolution. Scaffold porosity was created using the salt leaching method, in which an equivalent amount of pure NaCl microparticles (as a porogen) was added to the dissolved PEVAV/β-TCP solution. The resulting thick suspension was poured into a round steel mold measuring 5 mm in diameter and 1.5 mm in thickness and left to dry overnight at room temperature (25 °C). To further remove any solvent trace, the casted samples were placed in a vacuum oven for 24 h at 40 °C. NaCl particles were removed by immersion of the obtained samples in distilled water for 24 h, which was renewed every 4 h. The porous samples were placed again in the vacuum desiccator to form the pore connections based on the high-pressure difference applied to the pores walls [16]. All scaffolds were sterilized by γ-irradiation before implantation.

The properties of the PEVAV/β-TCP biocomposite are in brief came as the following, X-ray diffraction (XRD) revealed that the incorporation of β-TCP particles into the mixture had a mutual effect with the PDVAL component; moreover, the structure of PEVAL persisted unchanged. Scanning electron microscopy (SEM) showed a homogenous distribution of β-TCP particles in the polymeric matrix in both 50 and 70 wt%, with the resulted interconnected pores. These were confirmed by micro-CT assessment of total porosity of 48.53% and average pore size ranging from 200 to 400 µm, mechanical testing of the prepared PEVAV/β-TCP biocomposite yield a compressive strength with an average of 6.65 MPs. The detailed synthesis and physicochemical, morphological, and mechanical in vitro characterization of the new PEVAV/β-TCP biocomposite have been described previously [14].

### 2.2. Ethical Approval, Sample Size, Randomization, and Grouping of the Study Animals

The experimental protocol was ethically approved by the College of Dentistry Research Centre (CDRC), King Saud University, Riyadh, Saudi Arabia (Ref. # PR0101). The experimental animal model consisted of 40 healthy, skeletally mature male Wistar albino rats with an average age of 11 ± 2 weeks and ranging in weight from 350 to 450 g. Under daily certified veterinary monitoring, animals were acclimatized and housed (2 animals per rodent cage) in a controlled laboratory environment (temperature 22–24 °C; humidity, 45–55%; 12 h light/dark cycles; and *ad libitum* access to food and water). The experimental surgical procedures and laboratory animal care measures were performed according to the National Institutes of Health guidelines (NIH Publication #85-23 Rev.1985) [3]. the animals were randomly divided into one of the four groups using a random allocation software based on the biomaterial used for the regeneration of the critical-size defect (CSD) [17] (Table 1). Animals were tagged using a unique identifier code in a digital microchip that was injected into the animal via a subcutaneous route (Pet-ID Microchips Ltd. Saint Helena, UK.

### 2.3. General Anesthesia and Surgical Procedure

**Figure 1:**
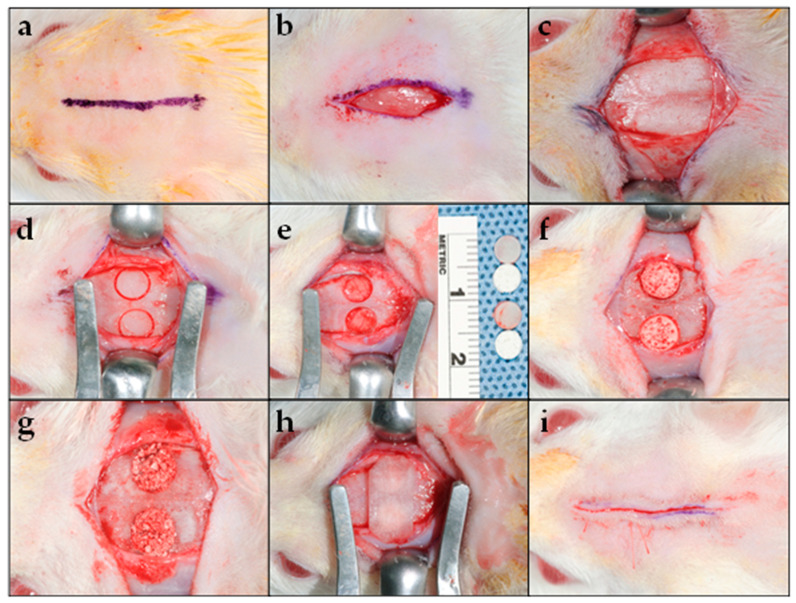
Surgical procedure: (**a**) Straight midline marking (15 mm in length) along the sagittal suture over the scalp’s parietal bone for the cutaneous incision; (**b**) linear cutaneous incision; (**c**) reflection of the skin and underlying tissues, including the musculature and the periosteum bilaterally, to expose the calvarium; (**d**) creation of a full-thickness critical-size defect on the parietal region bilaterally to the sagittal suture using a trephine drill with an outer diameter of 5 mm; (**e**) full-thickness bone (including the outer and inner cortices) was carefully removed, to prevent damage to the dura; (**f**) placement of the biocomposite scaffold or (**g**) β-TCP mixed with saline into the experimental defect; (**h**) RCM was placed over the defect; and (**i**) flap repositioning and closure using resorbable sutures.

All surgical procedures were carried out under general anesthesia. General anesthesia was achieved by intraperitoneal injection of a mixture containing xylazine hydrochloride (10 mg/kg of body weight; Xilagesic^®^, Laboratorios Calier S.A., Barcelona, Spain) and ketamine hydrochloride (50 mg/kg of body weight; HIKMA Pharmaceuticals, Amman, Jordan). The anesthetic drugs were titrated based on the animal’s weight, mixed, and administered as a single dose 15–20 min before starting the surgical procedure [3]. Anesthetized rats were placed in a prone position on the operating table. After shaving, the operation site (calvaria) was disinfected using povidone (7.5%) iodine solution (Avalon Pharma MECP, Riyadh, Saudi Arabia). A straight sagittal midline cutaneous incision (approximately 15 mm in length) was made on the animal’s scalp alongside the sagittal suture on top of the parietal bone. Skin, temporalis muscles, and the periosteum were reflected bilaterally in layers, exposing the calvarium. Using a trephine drill (5 mm outer diameter; Hee Sung Corp., Seoul, Korea) under constant irrigation with saline, two symmetrical bicortical round bony defects were created. Full-thickness bone was detached without damaging the dura. The defects were grafted with the biomaterial depending on the group to which the animal was randomly assigned. Next, the flaps were repositioned and sutured with 4–0 vicryl (Ethicon Coated VICRYL, Johnson & Johnson, Somerville, New Jersey, USA) [18]. Vital signs, such as respiratory rate and pattern, were monitored intra-operatively and post-operatively until the animals recovered from the general anesthesia. Post-operatively, the surgical sites were disinfected with povidone (7.5%). Immediately after surgery, a single intramuscular injection of antibiotic (0.0015 mL/g of body weight; Betamax, Nrborook Laboratories Ltd., Northern Ireland, UK) and analgesic (0.005 mL/gm of body weight; Analgivet 500, Can Tho City, Vietnam) were administered to all animals (Figure 1). All surgical procedures were performed under veterinary supervision by the same surgeon, who was blinded to the type of biomaterial used in the test groups. Animals were euthanized with CO_2_ inhalation at 4 and 10 weeks post-surgery following the American Veterinary Medical Association (AVMA) guidelines [19].

### 2.4. Micro-CT Analysis

**Figure 2:**
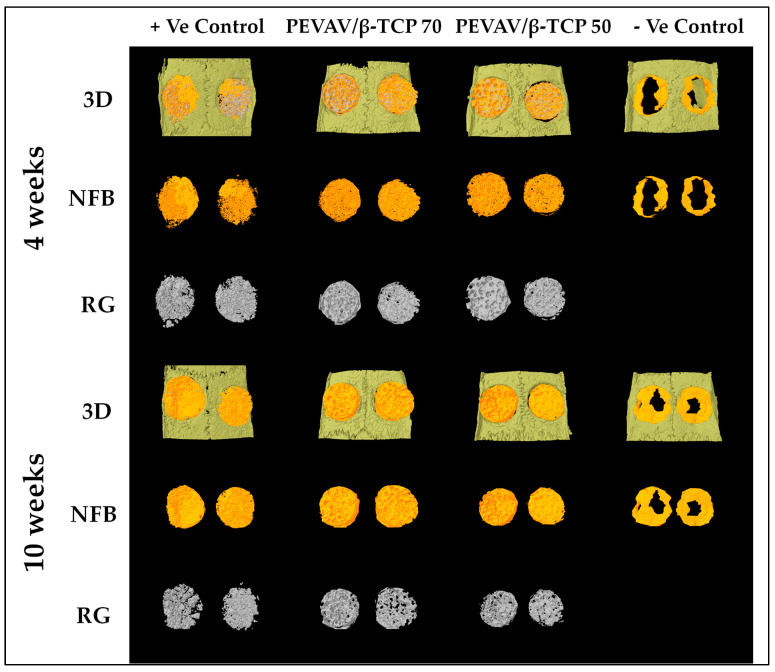
Reconstructed three-dimensional micro-CT images showing new bone formation (orange color) and remaining graft particles (gray color), within the critical size calvarial defect at 4 and 10 weeks. 3D; three dimensional, NFB; newly formed bone, RG; remaining graft.

The collected rat calvarias were scanned with a micro-CT scanner (SkyScan 1172; Bruker SkyScan, Kontich, Belgium). To prevent sample dehydration, the rat calvaria was wrapped in a transparent plastic film during the scanning. Digital micro-radiographic images were acquired at 50 Kv voltage, 98 µA current, 700 ms exposure time, 22 µm image pixel size, 1.0 Al filter, and d0.4 rotation step for 360° angle scanning parameters. Three dimensional image stacks were reconstructed using N-Recon^®^ (Bruker SkyScan, Kontich, Belgium) with a ring artifact reduction of 6, 25% beam hardening correction, and smoothing of 2 using an Asymmetrical boxcar. Two known density calcium hydroxyapatite (CaHA) phantoms with a predetermined mineral density (200 mg/cm^3^) were used to perform densitometric calibrations according to the manufacturer’s instruction protocol for CT attenuation in Hounsfield units (HU), to accurately calculate the newly formed bone mineral density (NFBMD) in the CTAn^®^ (Bruker SkyScan, Kontich, Belgium). CaHA phantoms were scanned and reconstructed in the same machine using the same parameters as those applied for the rat calvarias. The DataViewer^®^ (Bruker SkyScan, Kontich, Belgium) Software was used to determine uploaded reconstructed image quality and to reorient, resize, and resave data. The 2D/3D images were identified and analyzed using CTAn^®^ (Bruker SkyScan, Kontich, Belgium). The whole calvarial defect was measured as the region of interest and isolated using manually drawn contouring. NFB and remaining graft volume (RGV) were assessed in each image section based on HU values, in addition to visual differences between the two. The optimal threshold from the image histograms was determined, to exclude the RGV from the NFB with a separate micro-CT attenuation range for each. By multiplying the areas of NFB and RGP in each section by the slice section, the volume of NFB (NFBV) and RGV (RGV) were calculated. NFBMD was obtained using a similar technique. A trained micro-CT technician performed the scanning and the resulting data were interpreted by three independent observers. In addition to being standardized for inter-observer and intra-observer reliability and validity, the observers were blinded to animal grouping. Finally, CTVol^®^ (Bruker Skyscan, Kontich, Belgium) was used for 3D visualization and the production of color-coded images of the rat calvarias (Figure 2).

### 2.5. Statistical Analysis

Eighteen defects per group were estimated based on a statistical power of 0.90, a confidence level of 95%, and statistical significance (α) assumed at 0.05 (Epi Info StatCALC 7.0, CDC, Atlanta, GA, USA). However, a two-defect overestimation was considered in each group for any experimental losses. As two experimental calvarial defects were made in each animal, the final sample size of the study was 40 animals (n = 10 per group). The results pertaining to quantitative variables, namely NFBV, NFBMD, and RGV, were evaluated using the SPSS statistical package (Version 20, IBM Statistics, Chicago, IL, USA) and expressed as means ± standard deviations. One-way analysis of variance (ANOVA) with Tukey’s post hoc test at a level of significance of *p* < 0.05 was used for the comparison of outcomes between the groups at different time points.

## 3. Results

### 3.1. General Observations and Scaffold Applicability during Surgeries

During all surgeries, the biocomposite scaffolds were relatively rigid at room temperature and, after being soaked in saline, allowed easy manipulation and application to the surgical site. Moreover, the scaffolds exhibited an acceptable form and were stable during handling and adaptation to the surgical defects. No particle loosening from the scaffold was observed. The visual evaluation of the scaffold after implantation revealed that it filled the defect, and that scaffold architecture was maintained. An uneventful postoperative period was observed in all animals; no gross signs of adverse tissue reaction, biocomposite scaffold extrusion, infection, or inflammation were recorded.

### 3.2. Micro-CT Analysis

The mean values and standard deviations of the study variables (NFBV, NFBMD, and RGV) and the differences between them among all groups at the study time points are presented in Table 2.

#### 3.2.1. Comparison between the Groups in Terms of Newly Formed Bone Volume

Micro-CT analysis showed that, by the fourth week after grafting, the newly formed bone volume was higher in the PEVAV/β-TCP 70 group, followed by the positive control and the PEVAV/β-TCP 50 groups, i.e., 2.56 ± 0.52, 2.37 ± 0.33, and 2.01 ± 0.32 mm^3^, respectively, with a statistically significant difference observed only in the PEVAV/β-TCP 50 group. Conversely, the amount of NFBV (1.31 ± 0.28 mm^3^) was significantly lower in the negative control group vs. all other groups.

**Figure 3:**
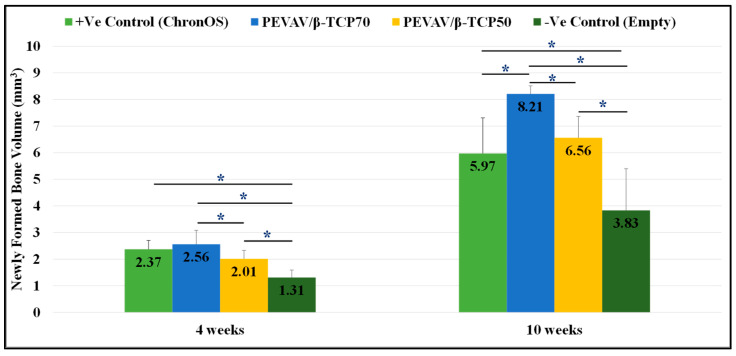
Bar graph presenting the changes in volume of newly formed bone in mean and standard error for all study groups at 4 weeks and 10 weeks. * Statistically significant difference (*p*-value < 0.05).

By the 10th week after grafting, the amount of NFBV was significantly higher in the PEVAV/β-TCP 70 group (8.21 ± 0.30 mm^3^) compared with all other groups. However, the NFBV of the PEVAV/β-TCP 50 group (6.56 ± 0.80 mm^3^) was higher than that of the positive control group (5.97 ± 1.34 mm^3^), albeit with no significant differences. Again, the NFBV was significantly lower in the negative control group vs. all other groups (3.83 ± 1.57 mm^3^) (Figure 3).

#### 3.2.2. Comparison between the Groups in Term of Newly Formed Bone Mineral Density

It was observed that, at the fourth week after grafting, the NFBMD was 1.29 ± 0.31 g/mm^3^, 1.10 ± 0.55 g/mm^3^, 0.92 ± 0.20 g/mm^3^, and 0.91 ± 0.30 g/mm^3^ in the positive control, PEVAV/β-TCP 70, PEVAV/β-TCP 50, and negative control groups, respectively. No statistically significant difference was observed between the groups in terms of NFBMD by week 4.

**Figure 4:**
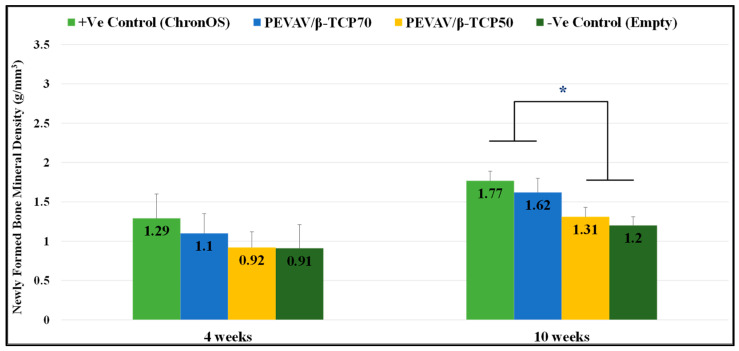
Bar graph presenting the changes in mineral density of newly formed bone in mean and standard error for all study groups at 4 weeks and 10 weeks. * Statistically significant difference (*p*-value < 0.05).

In contrast, the NFBMD was increased by the 10th week after grafting in all groups. The positive control showed the highest values of NFBMD, at 1.77 ± 0.12 g/mm^3^, with no significant differences from the PEVAV/β-TCP 70 group (1.62 ± 0.18 g/mm^3^); however, both groups (positive control and PEVAV/β-TCP 70) exhibited a significantly higher NFBMD compared with the PEVAV/β-TCP 50 (1.31 ± 0.12 g/mm^3^) and the negative control (1.20 ± 0.11 g/mm^3^) groups (Figure 4).

#### 3.2.3. Comparison between the Groups in Terms of Remaining Graft Volume

Regarding the volume of the remaining graft material, micro-CT scanning revealed that all grafted defects had a remnant of graft material by the 4th week, with no significant differences between them. Furthermore, defects in the positive control (grafted with ChronOS^®^) showed a lower amount of remaining graft volume (8.63 ± 0.88 mm^3^), followed by defects in the PEVAV/β-TCP 70 and PEVAV/β-TCP 50 groups (8.86 ± 1.35 and 9.32 ± 0.44 mm^3^, respectively).

**Figure 5:**
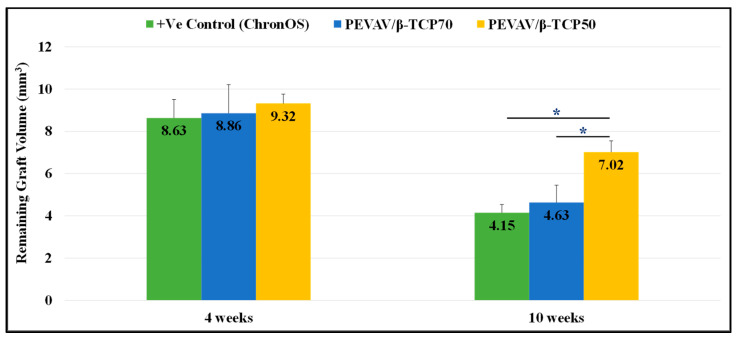
Bar graph presenting the changes in volume of remaining graft in mean and standard error for all study groups at 4 weeks and 10 weeks. * Statistically significant difference (*p*-value < 0.05).

By week 10, defects in the PEVAV/β-TCP 50 group exhibited a slight reduction in the amount of graft remnant volume (7.02 ± 0.53 mm^3^); however, this remained the highest compared with the PEVAV/β-TCP 70 and the positive control (ChronOS^®^) groups (4.63 ± 0.82 and 4.15 ± 0.39 mm^3^, respectively), with a statistically significant difference (Figure 5).

## 4. Discussion

The presented study was conducted to investigate whether the biocomposite scaffold had in vivo potential to act as an osteoconductive bone scaffold in calvarial critical-size defects of a rat model, as assessed using micro-CT. Furthermore, we evaluated the bone regeneration efficacy of the biocomposite scaffold in comparison with a commercially available bone graft material.

The NFBV in the PEVAV/β-TCP 70 group was the highest among all groups, with statistical significance detected at the 10th week. This agreed with the findings of Ignjatović et al. in his experimental study of a rat model aimed at assessing the osteoconductivity of a bone scaffold composed of hydroxyapatite (HAP)/chitosan (Ch)/poly-d,l-lactide-co-glycolide HAP/Ch-PLGA and comparing it with HAP, HAP/Ch, and a control group. At 4 weeks after transplantation, the defects grafted with the HAP/Ch-PLGA composite scaffold were filled with newly formed bone tissue, and further histological assessment showed an increase in osteoblast proliferation and size at the surface of the HAP/Ch-PLGA polymer blend compared with the other study groups [20]. The amount of β-TCP in the composite scaffold has been reported to play a role in its efficacy in bone regeneration. In 2010, van der Pol et al. conducted a study to assess the osteoconductivity of a PLLA/β-TCP composite scaffold in a large animal model (sheep) and using longer study periods (2, 4, and 12 months). Those authors found that the ratio of bone volume to tissue volume was higher in the commercially available graft material (ChronOS^®^) during the whole study period, which was in contrast with the result presented here. However, the authors suggested that these findings could be attributed to the higher ratio of PLLA (95 wt%) to β-TCP (5 wt%) in the scaffold [21]. The same reasoning could justify the findings obtained in the present study, i.e., the PEVAV/β-TCP 70 group, with the largest amount of β-TCP in the scaffold (PEVAV 30/β-TCP 70), resulted in the highest newly formed bone volume compared with other groups.

Micro-CT has become a well-known, noninvasive, three-dimensional assessment tool for bone regeneration in both experimental and preclinical studies [22]. Having the ability to measure the quantity of bone and its quality, micro-CT started to challenge traditional histological methods in the evaluation of osseous tissue regeneration [23,24,25]. To reflect the strength and mineralization of newly formed bone, newly formed mineral density was evaluated in the present experiment. The positive control defects, which were grafted with commercially available graft material (ChronOS^®^), showed the highest values of NFBMD. However, defects in the PEVAV/β-TCP 70 group showed comparable results to the positive control group, unlike defects in the PEVAV/β-TCP 50 group, which showed significantly lower values of NFBMD. These results agreed with the findings of an experimental study conducted in 2010 by Cao and Kuboyama. That study investigated the bone repairability of a biodegradable composite scaffold of PGA/β-TCP (1:1 and 1:3 weight ratios) in a rat model using micro-CT at 0, 14, 30, and 90 days after surgery. The mineral density was correlated with the amount of β-TCP, as defects grafted with PGA/β-TCP (1:3) scaffold had a higher NFBMD than did those grafted with the PGA/β-TCP (1:1) scaffold [26]. This was attributed mainly to the content of β-TCP (or the level of Ca and P) in the scaffold, which was most probably the same in our study.

Several studies have reported the effect of β-TCP in inducing cellular proliferation, osteogenesis, and the adsorption of proteins and growth factors, which consequently aids in bone regeneration [4,27,28,29]. However, β-TCP has the disadvantage of rapid degradation, thus restricting its ability to promote bone regeneration over an extended time [2].

Hence, the combination of β-TCP with a biodegradable polymer provides operational freedom to surgeons, to costume design the scaffold according to the osseous defect [15]. This method favorably aids in bone regeneration and mineralization through the controlled, steady degradation of TCP, with its subsequent ceramolysis providing calcium ions for the bone mineralization process [30]. This was observed in the results of the presented study, in which RGV was lower in the defects of the positive control group, which were grafted with pure β-TCP (ChronOS^®^), compared with defects grafted with biocomposite scaffolds of either PEVA/β-TCP 70 or 50.

The importance of RCM in bone regeneration cannot be overstressed; it increases chemotaxis of fibroblasts and supports osteogenesis via osteoblasts adhesion to its surface. This study elected to use RCM to aid in graft particulate stabilization (ChronOS^®^) by achieving hemostasis around the graft particles by enhancing fibrin linkages and encouraging attachment of the platelet utilizing the well-known properties of RCM [31,32]. Consequently, provide the best conditions in the positive control because bicortical calvarial defect in the rat model is considered non-self-contained. Moreover, previous studies showed that NFBV and NFBMD in rat calvarial defects grafted with β-TCP and covered with RCM found to be superior to defects grafted with β-TCP without RCM [33].

Although, the use of RCM to cover bone defects in studies assessing bone regeneration using biocomposite scaffold is not consistent in the literature [15,25,34]. It was used in all groups in the presented study for standardization. Further studies are required to assess the role of RCM in bone regeneration of defects grafted with a biocomposite scaffold.

PEVAV polymer blend have been recently synthesized and tested only once as drug carrier system [13,35]. Up to our knowledge, it has never been added to any synthetic graft material nor been assessed for bone regeneration.

In this study, PEVAV was added to β-TCP using solvent casting/salt leaching technique with interconnected porosity. A similar technique has been cited in the literature; Sadiasa et al. used the same technique to fabricate PCL/ PLLA scaffold. SEM revealed the resulting interconnected pores with pore sizes ranging from 100 to 300 μm. Moreover, cell proliferation assays, gene expression, and new bone formations revealed a higher PLLA concentration blended into the scaffold (PR70:30 PCL:PLLA polymer ratio) [7]. Despite that the results of PEVAV/β-TCP 70 can be found marginal to the positive control (with higher NFBV and NFBMD in PEVAV/β-TCP 70 group). The addition of β-TCP to PEVAV polymer blend can enable the operator to modify its physiochemical properties, design the biocomposite scaffold according to the required shape and size; furthermore, it can be enhanced with biomolecules such as antimicrobial agents [14]. Which came in agreement with Miyai et al. Their results showed that the PCL/β-TCP composite scaffold loaded with gatifloxacin effectively controlled bone infection whenever implanted in the defects. In contrast with the defects treated with debridement only, osteomyelitis lesions were widely extended in the proximal−distal direction. Furthermore, new bone formation was observed in the scaffold defect interface at 4 weeks post-grafting [8].

## 5. Conclusions

According to the results of the present micro-CT study, bone regeneration using a PEVAV/β-TCP composite scaffold resulted in higher new bone formation compared with the control group by week 4 after transplantation. However, 70 wt% of β-TCP in the PEVAVE/β-TCP composite scaffold showed the highest (significant) newly formed bone and mineral density, with acceptable scaffold resorption. Within the limitations of this study, the PEVAV/β-TCP composite scaffold showed its potentiality for bone regeneration of critical-sized calvarial defects in a rat model. However, further studies will be essential to determine its clinical applicability and the biomechanical features of the newly formed bone.

## Figures and Tables

**Table 1:** Grouping of the study animals according to the material used in CSD grafting.

Study Group (n = 10/group)	Biomaterial Used in Grafting the CSD
**Positive control**	CSD was filled with β-TCP (ChronOS^®^, DePuy Synthes, Addison, TX, USA) soaked in normal saline and covered by RCM (BioCollagen; 0.2 mm × 5 mm × 7.5 mm; BIOTECK S.P.A., Vicenza, Italy).
**PEVAV/β-TCP 70**	CSD was filled with PEVAV/ β-TCP 70 biocomposite scaffold soaked in normal saline and covered by RCM (BioCollagen; 0.2 mm × 5 mm × 7.5 mm; BIOTECK S.P.A., Vicenza, Italy).
**PEVAV/β-TCP 50**	CSD was filled with PEVAV/ β-TCP 50 biocomposite scaffold soaked in normal saline and covered by RCM (BioCollagen; 0.2 mm × 5 mm × 7.5 mm; BIOTECK S.P.A., Vicenza, Italy).
**Negative control**	CSD was kept empty, to be filled with a blood clot and covered by RCM (BioCollagen; 0.2 mm × 5 mm × 7.5 mm; BIOTECK S.P.A., Vicenza, Italy).

CSD: critical size defect; RCM: resorbable collagen membrane; β-TCP: beta-tricalcium phosphate.

**Table 2:** Volume and mineral density parameters of the newly formed bone and remaining graft volume in the study groups at the two time points of the evaluation, presented as the mean ± standard deviation.

Time Point	Group (n = 10)	Newly Formed Bone Volume (mm^3^)	Newly Formed Bone Mineral Density (g/mm^3^)	Remaining Graft Volume (mm^3^)
4 weeks	Positive Control	2.37 ± 0.33 ″	1.29 ± 0.31	8.63 ± 0.88
	PEVAV/β-TCP 70	2.56 ± 0.52 ″§	1.10 ± 0.25	8.86 ± 1.35
	PEVAV/β-TCP 50	2.01 ± 0.32 ″^	0.92 ± 0.20	9.32 ± 0.44
	Negative Control	1.31 ± 0.28 †^§	0.91 ± 0.30	-
10 weeks	Positive Control	5.97 ± 1.34 ″^	1.77 ± 0.12 §″	4.15 ± 0.39 §
	PEVAV/β-TCP 70	8.21 ± 0.30 ″†§	1.62 ± 0.18 §″	4.63 ± 0.82 §
	PEVAV/β-TCP 50	6.56 ± 0.80 ^″	1.31 ± 0.12 †^	7.02 ± 0.53 †^
	Negative Control	3.83 ± 1.57 †^§	1.20 ± 0.11 †^	-

† Statistically significant difference compared with the positive control group; ^ Statistically significant difference compared with the PEVAV/β-TCP 70 group; § Statistically significant difference compared with the PEVAV/β-TCP 50 group; ″ Statistically significant difference compared with the negative control group.

## Data Availability

Data sharing not applicable.
